# The Effect of Cellular Stress on T and B Cell Memory Pathways in Immunized and Unimmunized BALB/c Mice*

**DOI:** 10.1074/jbc.M116.746057

**Published:** 2016-08-08

**Authors:** Yufei Wang, Durdana Rahman, Mukesh Mistry, Thomas Lehner

**Affiliations:** From the Mucosal Immunology Unit, King's College London at Guy's Hospital, London SE1 1UT, United Kingdom

**Keywords:** cellular immune response, heat shock protein (HSP), inflammasome, interleukin 1 (IL-1), oxidative stress, stress response, AID, IL-15, T and B memory cells

## Abstract

Immunological memory is a fundamental function of vaccination. The antigenic breakdown products of the vaccine may not persist, and undefined tonic stimulation has been proposed to maintain the specific memory. We have suggested that cellular stress agents to which the immune cells are constantly exposed may be responsible for tonic stimulation. Here we have studied four stress agents: sodium arsenite, an oxidative agent; Gramicidin, eliciting K^+^ efflux and calcium influx; dithiocarbamate, a metal ionophore; and aluminum hydroxide (alum), an immunological adjuvant. The aims of this study are to extend these investigations to T and B cell responses of unimmunized and ovalbumin (OVA)-immunized BALB/c mice, and furthermore, to ascertain whether stress is involved in optimal expression of memory B cells, as demonstrated in CD4^+^ T cells. Examination of the homeostatic pathway defined by IL-15/IL-15R (IL-15 receptor) interaction and the inflammasome pathway defined by the IL-1-IL-1R interaction between dendritic cells (DC) and CD4^+^ T cells suggests that both pathways are involved in the development of optimal expression of CD4^+^CD45RO^+^ memory T cells in unimmunized and OVA-immunized BALB/c mice. Furthermore, significant direct correlation was found between CD4^+^CD44^+^ memory T cells and both IL-15 of the homeostatic and IL-1β of the inflammasome pathways. However, CD19^+^CD27^+^ memory B cells *in vivo* seem to utilize only the IL-15/IL-15R homeostatic pathway, although the proliferative responses are enhanced by the stress agents. Altogether, stress agents may up-regulate unimmunized and OVA-immunized CD4^+^CD44^+^ memory T cells by the homeostatic and inflammasome pathways. However, the CD19^+^CD27^+^ memory B cells utilize only the homeostatic pathway.

## Introduction

Basal homeostatic CD4^+^ memory T cells are maintained by tonic T cell receptor (TCR)^2^ signaling, below the threshold to induce overt activation (1–4). Memory T cells do not require TCR-MHC antigen-driven interaction to survive, as was reported with microorganisms (5, 6) and highlighted with smallpox (7). The homeostatic dendritic cell (DC)-CD4^+^ T cell interactive memory circuit can be stimulated by heat, oxidative or K^+^ efflux, and Ca^2+^ influx stress agents. They induce reactive oxygen species (ROS) in DC, leading to NFκB signaling and membrane-associated (ma)IL-15 expression (8, 9). maIL-15 ligates IL-15R complex on CD4^+^ T cells, activating Jak3 and STAT5 phosphorylation signaling pathway to induce CD40L expression, T cell proliferation, and IFN-γ production (9). CD40 ligand on CD4^+^ T cells in turn re-activates CD40 molecules on DC, inducing DC maturation and IL-15 expression, thereby maintaining a feedback circuit. The proliferating CD4^+^ T cells were characterized as CD45RA^−^CD62L^+^ central memory cells, which undergo homeostatic proliferation (9). These data were confirmed by *in vivo* murine experiments (10) and extended to Gramicidin, a potassium-releasing antibiotic (11), which functions as an ionophore, penetrating cell membranes and causing K^+^ efflux (12), and is effective against Gram-positive bacteria and viruses. It has been used clinically as an ophthalmic antimicrobial agent. Sodium arsenite is an oxidative stress agent releasing free radicals of ROS, which leads to a state of redox disequilibrium (13) Dithiocarbamate is a metal ionophore, which functions as a fungicide (14) and is used in agriculture. The results suggested that stress agents utilize a dual signaling pathway mediated by the interaction between DC and CD4^+^ T cells. The homeostatic (H) pathway activates NFκB, which transactivates maIL-15 expression on DC, binding IL-15R on CD4^+^ T cells and inducing CD40L expression (9). Recently, we have presented evidence *in vitro* in primary human T cells that both the homeostatic (H) and inflammasome (I) pathways are required for optimal CD4^+^CD45RO^+^ memory T cell expression (15).

The objectives of this study were to study the effect of three stress agents and alum, an adjuvant, which also demonstrates stress-mediated functions in DC interacting with CD4^+^ T and CD19^+^ B cells, to induce T cell receptor-independent homeostatic memory in CD44^+^ memory T cells and CD27^+^ memory B cells in BALB/C mice (9, 10). The phenotypic expression of memory T and B cells and their proliferative responses were then compared with the effect of the same stress agents, but in OVA-immunized mice. Thus, both unimmunized and OVA-immunized memory T and B cells were evaluated with reference to the H and I pathways. The results suggest that although the H and I pathways are required to elicit optimal CD4^+^CD44^+^ memory T cells in both unimmunized and OVA-immunized studies, CD19^+^CD27^+^ memory B cells used only the H pathway. The specificities of the stress-treated, unimmunized T and B memory cells were not evaluated, but they are likely to represent the steady state of memory responses to the past exposure of multiple antigens, as suggested for prior immunization with tetanus toxoid in human T cell proliferation (9).

## Results

To study DC and T and B cell responses and functions induced by stress, we used unimmunized and OVA-immunized BALB/c mice. Splenic CD11c^+^ DC, naive and memory CD4^+^ T cells, and CD19^+^ B cells were studied for their responses to stress, the role of H and I pathways, as well as the effect on activation-induced deamination (AID) and on IgG, IgM, and IgA antibodies.

### 

#### 

##### The Effect of Stress Agents on Splenic CD11c DC in Unimmunized and OVA-immunized BALB/c Mice

**FIGURE 1.**
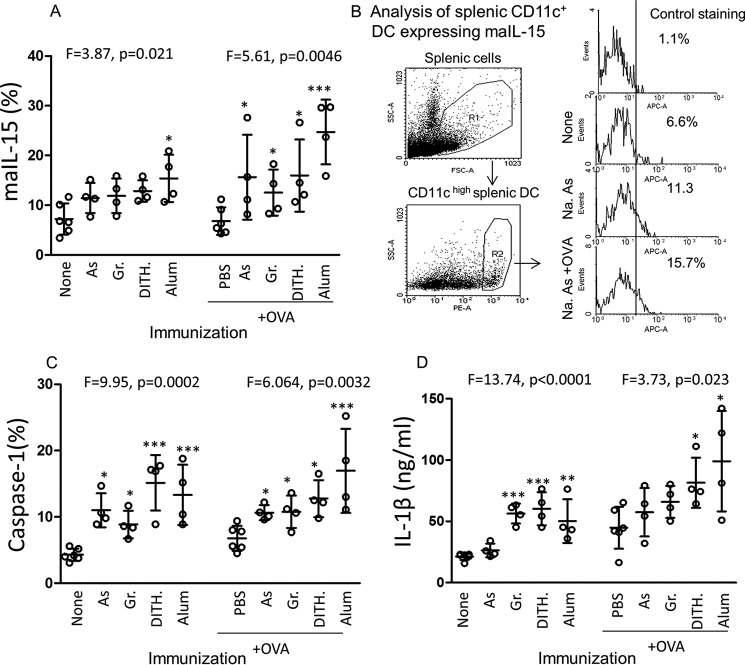
**Expression of maIL-15, activated caspase-1, and IL-1β in mouse splenic CD11c^+^ DC, following treatment in BALB/c mice with stress agents without or with OVA.** *A–D*, maIL-15 expressed in percentages (*A*), gating strategy and representative FACS illustration (*B*), caspase-1 in percentages (*C*), and IL-1β in ng/ml (*D*), all following *in vivo* treatment of BALB/c mice with sodium arsenite (*As*), Gramicidin (*Gr*), dithiocarbamate (*Dith.*), and alum. All were determined by specific mAb using FACS, except Caspase-1 was determined by a kit and IL-1β was determined by ELISA. All data are expressed as mean ± S.D. ANOVA (*F* value) was used to determine significance between each group of 4 treated and the untreated control and the paired *t* test within the groups; *n* = 4–6 mice per group. *, *p* < 0.05, **, *p* < 0.01, ***, *p* < 0.001.

We have previously demonstrated that maIL-15 and IL-1β are up-regulated in CD11C^+^ splenic DC when BALB/c mice were treated with stress agents and OVA (10). We hypothesized from our studies with CD4^+^ T cells (15) that the homeostatic pathway is driven by interaction between maIL-15DC and IL-15Ra on B cells, whereas the inflammasome pathway is driven by interaction between IL-1 expressed by DC and IL-1R on B cells. Analysis of variance of maIL-15 in splenic CD11C^+^ DC showed significant difference between the stress agent-treated mice without OVA immunization (*F* = 3.868, *p* = 0.021), although separately only alum reached significance (Fig. 1*A*). A greater increase in maIl-15 was observed in DC from the stress agents in OVA-immunized mice (*F* = 5.61, *p* = 0.004) and separately with each stress agent (Fig. 1*A*). The gating strategy and representative flow cytometry are presented in Fig. 1*B*.

Caspase-1 was very significantly up-regulated, in both the untreated (*F* = 9.947, *p* = 0.0002) and OVA-immunized animals (*F* = 6.064, *p* = 0.0032, Fig. 1*C*). Activated caspase-1 converts pro-IL-1 into IL-1β, which was similarly up-regulated by the stress agents, surprisingly to a greater extent in the untreated mice (*F* = 13.74, *p* < 0.0001) than in the OVA-immunized mice (*F* = 3.734, *p* = 0.023, Fig. 1*D*). Thus, CD11c^+^ DC were activated by the stress agents to initiate the H and I pathways, in both unimmunized and OVA-immunized BALB/c mice, although significant differences were found between the stress agents.

##### The Effect of Stress on CD40L Expression in CD4^+^ T Cells

**FIGURE 2.**
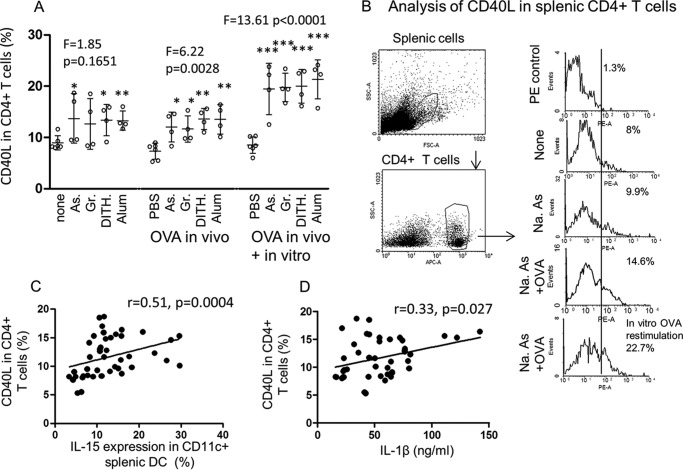
**CD40L expression in CD4^+^ T cells and correlations between CD40L and IL-15 or IL-1β.** *A*, CD40L in CD4^+^ T cells was studied by flow cytometry, following treatment with the four stress agents, without or with OVA *in vivo*, and another group was re-treated with OVA *in vitro* for 3 days (*n* = 4–6/group). *As*, sodium arsenite; *Gr*, Gramicidin; *Dith.*, dithiocarbamate. *B*, gating strategy and illustration of flow cytometry. *Na. As*, sodium arsenite. *C* and *D*, correlations were tested between CD40L and maIL-15 (*C*) and IL-1β (*D*), using all animals (*n* = 40). All data are expressed as mean ± S.D. Statistical analysis was carried out as in Fig. 1, and the Spearman correlation test was used in *C* and *D*. *, *p* < 0.05, **, *p* < 0.01, ***, *p* < 0.001.

Previous studies have shown that CD40L is increased with antigen or mitogen stimulation (16), and we have demonstrated that stress may also up-regulate CD40L in the absence of direct antigen stimulation (10). Here we have investigated *in vivo* any difference in response to stress in unimmunized as compared with OVA-immunized CD40L response. All four stress agents induced an increase of CD40L in the unimmunized group, but the ANOVA was not significant, unlike comparison between each stress agent (except Gramicidin) and the baseline control (Fig. 2*A*). This discrepancy is most likely to be due to the large S.D. of CD40L induced by two of the stress agents. However, the OVA-dependent responses were significantly up-regulated (*F* = 6.22, *p* = 0.0028) and further boosted on *in vitro* re-stimulation with OVA (*F* = 13.61, *p* < 0.0001, Fig. 2*A*). Gating of CD4^+^ T cells and the effect of stress agents are shown by flow cytometry (Fig. 2*B*). We then studied CD40L expression to find out whether it was dependent on H pathway, I pathway, or both pathways. Statistical analysis of any correlation utilized data from all 40 animals treated with the four stress agents *in vivo* (excluding those *in vitro* treated with OVA). The aim was to analyze the biological effect of cellular stress between DC generating maIL-15 of the homeostatic and IL-1β of the inflammasome pathway with CD40L expressed by CD4^+^ T cells. A significant direct correlation was found between CD40L in CD4^+^ T cells with both IL-15 (*p* = 0.0004, Fig. 2*C*) and IL-1β (*p* = 0.027, Fig. 2*D*) expressed by the CD11c DC. This is consistent with the concept that both pathways are involved in activating CD4^+^ T cells, although the homeostatic pathway appears to be stronger than that of the inflammasome. Comparison of CD40L expression with IL-15 and IL-1β in Fig. 1, *A* and *C*, showed no significant difference on further statistical analysis of unimmunized and OVA-immunized mice (data not shown).

##### Transcription Factors in Murine CD4^+^ T Cells

**FIGURE 3.**
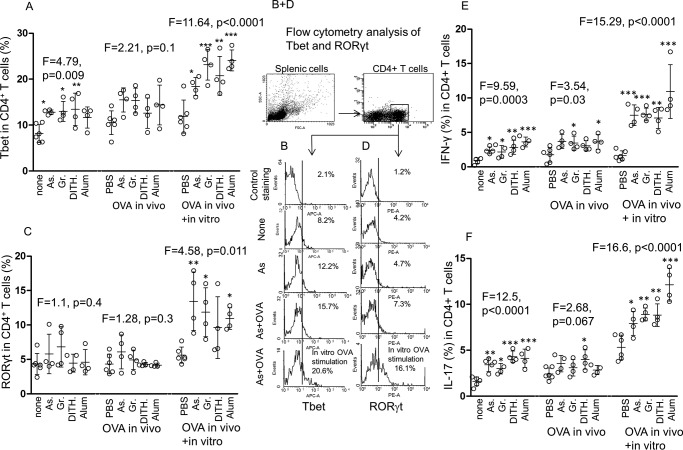
**Expression of Tbet and RORγt transcription factors and the corresponding IFN-γ and IL-17 cytokines in unimmunized and OVA-immunized, stress agent-treated CD4^+^ T cells.** *A*, the effect of stress agents in unimmunized, OVA-immunized, and *in vitro* OVA re-stimulated CD4^+^ T cells on Tbet expression. *As*, sodium arsenite; *Gr*, Gramicidin; *Dith.*, dithiocarbamate. *B*, gating strategy and representative FACS illustration. C and D, as in *A* and *B* for RORγt expression. *E* and *F*, IFN-γ in CD4^+^ T cells (*E*) and IL-17 in CD4^+^ T cells (*F*), both in the same cohorts as in *A*. All data are expressed as mean ± S.D. *, *p* < 0.05, **, *p* < 0.01, ***, *p* < 0.001.

The CD4^+^ T cell transcription factors were studied in splenic cells from BALB/c mice treated with the stress agents, to differentiate between unimmunized and OVA-immunized activation of transcription factors. A significant increase in Tbet expression was found in CD4^+^ T cells (*F* = 4.792, *p* = 0.009) with all stress agents except alum (*p* < 0.05 to *p* < 0.01, Fig. 3*A*). The OVA-immunized, stress-treated mice also showed an increase in Tbet, but this did not reach statistical significance (*F* = 2.21, *p* = 0.1) unless the cells were re-stimulated *in vitro* with OVA (*F* = 11.64, *p* < 0.0001 and individually *p* < 0.05 to *p* < 0.001, Fig. 3*A*). The representative FACS data are presented in Fig. 3*B*. RORγt transcription in CD4^+^ T cells failed to show a significant increase in the unimmunized stress-stimulated DC, but the OVA-immunized re-stimulated cells were significantly up-regulated (*F* = 4.58, *p* = 0.011 or individually *p* < 0.05 to *p* < 0.01; Fig. 3*C*); the FACS data are shown in Fig. 3*D*. Gata3 failed to show significant changes with the stress agents (data not shown). The results suggest that although Tbet is up-regulated in both unimmunized and OVA-dependent stress-treated mice, RORγt is up-regulated only in the stress-treated, OVA-immunized, and boosted animals. These transcription data are consistent with Th1 and Th17 but not Th2 responses as shown below.

##### The Effect of Stress Agents on Induction of Intracellular Cytokines

Stress-induced H and I pathways elicited up-regulation of Tbet, which transcribes IFN-γ, and RORγt, which transcribes IL-17 cytokines, but GATA3, which mediates IL-4 and IL-10, was not increased, except when IL-4 was treated with alum. Significant up-regulation of IFN-γ was found in the CD4^+^ T cells of unimmunized animals with all stress agents (*F* = 9.59, *p* = 0.0003, Fig. 3*E*). However, OVA-immunized mice showed limited although significant up-regulation of IFN-γ (*F* = 3.54, *p* = 0.03) with sodium arsenite (*p* < 0.05) and alum (*p* < 0.05 Fig. 3*E*), which was greatly boosted with OVA when treated with all stress agents (*F* = 15.29, *p* < 0.0001, Fig. 3*E*). Similar results were found on analysis of the IL-17 data, with significant up-regulation by all stress agents in the OVA-unimmunized animals (*F* = 12.5, *p* < 0.0001). OVA-immunized animals failed to show significant increase in IL-17 (*F* = 2.68, *p* = 0.067), but *in vitro* boosting with OVA greatly increased IL-17 production (*F* = 16.6, *p* < 0.0001; Fig. 3*F*). Neither IL-4 nor IL-10 showed any increase with the stress agents in CD4^+^ T cells from the unimmunized or OVA-immunized mice (with the exception of IL-4 with alum). These results are consistent with the transcription factors, showing significant up-regulation of Tbet and RORγt but not GATA3.

##### The Effect of Stress on CD44^+^ Memory CD4^+^ T Cells

**FIGURE 4.**
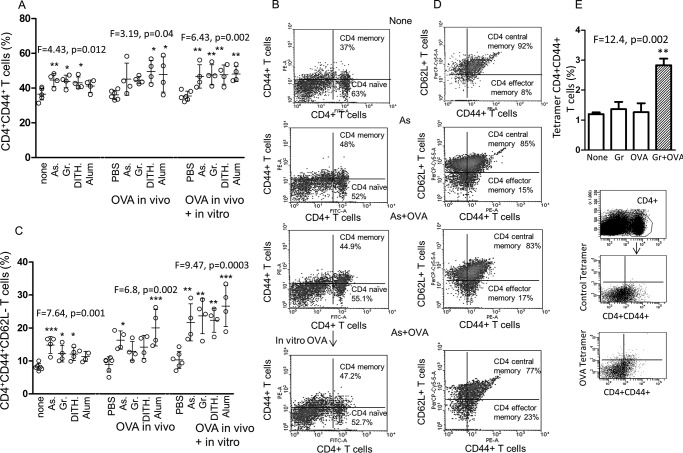
**Expression of CD4^+^CD44^+^ memory T cells and CD 62L^−^ effector memory T cells.** *A*, the effect of the stress agents without or with OVA immunization and the latter with OVA *in vitro* on CD4^+^CD44^+^ memory T cells. *As*, sodium arsenite; *Gr*, Gramicidin; *Dith.*, dithiocarbamate. *B*, representative FACS illustration. *C*, similar investigation in CD4^+^CD44^+^ CD62L^−^ effector memory T cells as in *A*, with representative FACS illustration (*D*). *E*, tetramer staining of OVA-specific CD4^+^CD44^+^ memory T cells in untreated animals and in animals treated with Gramicidin only (*Gr*), OVA only (*OVA*), and Gramicidin + OVA (*Gr*+*OVA*). All data are expressed as mean ± S.D. *, *p* < 0.05, **, *p* < 0.01, ***, *p* < 0.001.

The role of unimmunized *versus* specifically immunized memory T cell development is of fundamental importance to our understanding of maintenance of memory in the absence of antigen stimulation. The CD4^+^CD44^+^ memory T cells were significantly up-regulated with all stress agents, except alum (*F* = 4.432, *p* = 0.012, Fig. 4*A*). OVA-immunized mice also demonstrated significant increase with the stress agents (*F* = 3.189, *p* = 0.04, Fig. 4*A*), but this was limited to dithiocarbamate and alum. However, *in vitro* re-stimulation with OVA of the DC significantly boosted CD44^+^ memory CD4^+^ T cells (*F* = 6.431, *p* = 0.002), and each stress agent up-regulated these cells (*p* < 0.01, Fig. 4*A*). Representative FACS data are presented (Fig. 4*B*).

The effector memory CD4^+^CD62L^−^ T cells were then examined, and these were comparable, although greatly enhanced, as compared with the CD44^+^ memory T cells (Fig. 4*C*) in the unimmunized (*F* = 7.641, *p* = 0.0001), OVA-immunized (*F* = 6.798, *p* = 0.002), and boosted (*F* = 9.465, *p* = 0.0003) cells. Representative FACS data are presented (Fig. 4*D*). As expected, all unimmunized and OVA-immunized or OVA re-stimulated cells showed significant decrease in the CD4^+^CD44^+^CD62L^+^ central memory T cells (*F* = 7.641, *p* = 0.001, *F* = 6.798, *p* = 0.002 and *F* = 9.465, *p* = 0.0003, respectively, data not shown). The specificity of the CD4^+^CD44^+^ memory T cells to OVA was demonstrated by tetramer staining in Gramicidin-treated OVA-immunized cells and gating (*p* < 0.01, Fig. 4*E*). Overall the results are consistent with our previous findings, except that up-regulation of both CD4^+^CD44^+^ memory T cells and the corresponding CCR7^−^ effector memory cells is demonstrated not only in unimmunized but also immunized CD4^+^ T cells.

##### The Effect of Stress on CD4^+^ T Cell Proliferation

**FIGURE 5.**
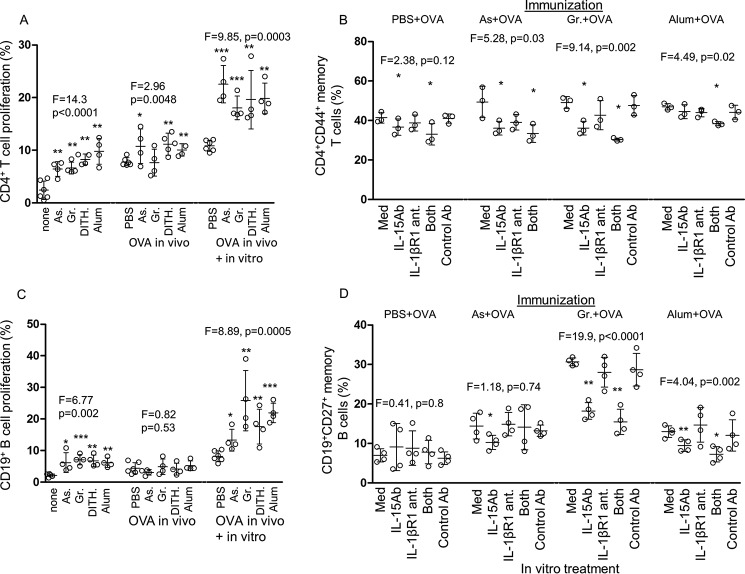
**Proliferation of CD4^+^ T and CD19 B cells; the role of H and I pathways in T and B memory cells tested by inhibition with IL-15 mAb and IL-1β R1 antagonist, respectively.** *A*, the proliferative responses in CD4^+^ T cells were studied in stress-treated unimmunized, OVA-immunized, and *in vitro* OVA re-stimulated groups of mice by the CFSE dye dilution method. *As*, sodium arsenite; *Gr*, Gramicidin; *Dith.*, dithiocarbamate. *B*, inhibitions of IL-15, IL-1 βR1, or both in CD4^+^CD44^+^ memory T cells of OVA-immunized mice, cultured *in vitro* for 5 days in the presence of mAb to IL-15 (10 μg/ml) or IL-1βR1 antagonist (50 m) (*n* = 4–6). CD4^+^ memory T cells were inhibited without stress treatment, and both inhibitors were involved when treated with the stress agents. *C*, CD19^+^ B cell proliferation was examined as in *A. D*, inhibition studies of CD19^+^CD27^+^ memory B cells as in *B*. All data are expressed as mean ± S.D. *Med*, medium.

The functional effect of stress on T cell proliferation was studied in splenic cells as above. CD4^+^ T cells showed significant up-regulation of proliferation with all stress agents in the unimmunized (*F* = 14.3, *p* < 0.0001, Fig. 5*A*) and OVA-immunized mice (*F* = 2.96, *p* < 0.005, except for Gramicidin), but when re-stimulated with OVA, they were all enhanced (*F* = 9.85, *p* < 0.0005, Fig. 5*A*). It seems that the stress agents enhance expansion of both unimmunized and OVA-immunized CD4^+^ T cells, but there was a great increase in the OVA-immunized baseline, without treatment with the stress agents, which accounted for the limited increase in the OVA-immunized animals.

##### The Role of Stress-induced H and I Pathways in CD4^+^CD44^+^ Memory T Cells

The aim of the inhibition studies of the H and I pathways by treatment with IL-15 antibodies and IL-1βR1 inhibitors, respectively, was to find out whether the CD4^+^CD44^+^ memory T cells require both pathways for optimum expression *in vivo*, as demonstrated for unstimulated human CD4^+^ memory T cells *in vitro* (14). Here we show in BALB/c mice that immunization with OVA increased the CD4^+^CD44^+^ memory T cells with Gramicidin and alum (*p* < 0.05, Fig. 5*B*), but not sodium arsenite (large S.D.). IL-15 antibody inhibited significantly with all except alum treatment, although maximum inhibition resulted when both IL-15 antibody and IL-1βR1 inhibitor were used, but the 5% level of significance with alum was reached only when both inhibitors were used (Fig. 5*B*).

##### The Effect of Stress on Unimmunized and OVA-immunized CD19^+^ B Cell Proliferation, as Well as on the H and I Pathways in CD19^+^CD27^+^ B Memory Cells

As with CD4^+^ T cells, CD19^+^ B cells in stress-treated mice showed significantly increased proliferation with all the stress agents in the unimmunized mice (*F* = 6.77, *p* = 0.002; Fig. 5*C*). However, the OVA-immunized stress-treated animals demonstrated a significant increase in B cell proliferation only if they were re-stimulated *in vitro* with OVA (*F* = 8.89, *p* = 0.0005, Fig. 5*C*). This difference was also seen with AID and suggests that B cells are required to be boosted with the antigen to express CD40L and proliferative responses. Inhibition studies of the H and I pathways were then carried out with CD19^+^CD27^+^ B memory cells to find out whether they require both pathways for optimum expression of memory B cells, as demonstrated with CD4^+^CD44^+^ memory T cells. Memory B cells showed significant increase with sodium arsenite and Gramicidin, but inhibition in OVA-immunized mice treated with the stress agents was limited to IL-15 antibodies, and that applied also to alum (*p* < 0.005 to *p* < 0.0001, Fig. 5*D*). When both inhibitors were used, there was no significant increase over that elicited by IL-15 antibodies alone. These studies suggest that unlike CD4^+^CD44^+^ memory T cells, the CD19^+^CD27^+^ memory B cells are only IL-15 (H) pathway-dependent in the interaction between DC and CD19^+^CD27^+^ B cells.

##### The Effect of Stress on Unimmunized and OVA-immunized Expression of IL-15Rα and IL-1RI in CD19^+^ Naive and CD27^+^ Memory B Cells

**FIGURE 6.**
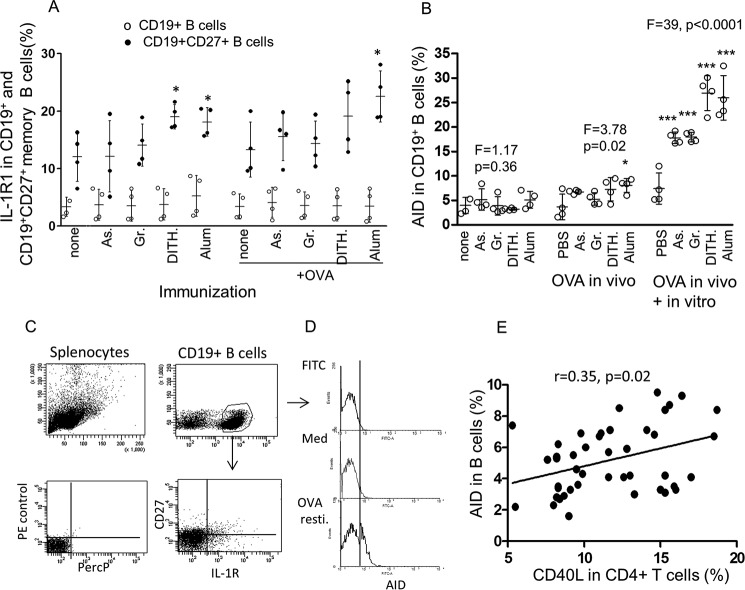
**Expression of IL-1R (in percentages) in CD19 naive and CD19^+^CD27^+^ memory B cells and AID in CD19^+^ B cells in BALB/c mice.** *A* and *B*, expression of IL-1R (in percentages) in CD19 naive and CD19^+^CD27^+^ memory B cells (*A*) and AID in CD19^+^ B cells (*B*), in unimmunized cultures, OVA-immunized cultures, and *in vitro* OVA re-stimulated cultures. *As*, sodium arsenite; *Gr*, Gramicidin; *Dith.*, dithiocarbamate. *C* and *D*, gating strategy and flow cytometry. *E*, correlation between CD40L in CD4^+^ T cells and AID in CD19^+^ B cells (*n* = 40). All data are expressed as mean ± S.D. *, *p* < 0.05, **, *p* < 0.01, ***, *p* < 0.001. *PercP*, peridinin-chlorophyll protein; *Med*, medium; *resti*, restimulation.

To elicit the H and I pathways in B cells, we studied expression of the corresponding receptors to the IL-15 and IL-1β expressed by the DC. A 6.5-fold increase in IL-15Rα and an ∼4-fold increase in IL-1RI expression were found in the memory CD27^+^ B cells, as compared with the naive CD19^+^ B cells (Fig. 6*A*; only IL-1R1 results are presented and gating in Fig. 6*B*). Although a small proportion of CD19^+^ B cells in unimmunized and OVA-immunized animals expressed IL-15Rα (3.5 ± 0.9 and 3.2 ± 0.2%) and IL-1R1 (3.5 ± 1.7 and 3.4 ± 2.2%), respectively, treatment with the stress agents failed to up-regulate these receptors (data not shown). However, because IL-15Rα and IL-1RI were greatly increased in CD27^+^CD19^+^ memory B cells, treatment with the stress agents showed significant increase only in the unimmunized, dithiocarbamate-, or alum-treated cells and in OVA-immunized cells treated only with alum, as compared with the CD27^+^-untreated controls (*p* < 0.05, Fig. 6*A*, only IL-1R1 presented).

##### The Effect of Stress on AID in CD19^+^ B Cells

AID is a fundamental function of B cells initiating somatic hypermutation (SHM), which generates high affinity antibodies by a process of affinity maturation (17–19). AID also elicits class switch recombination (CSR) of antibody isotypes from IgM to IgG, IgA, and IgE (17–19). AID in unimmunized stress-treated mice showed no significant change (Fig. 6*C*), but in the OVA-immunized mice, it was significantly increased (*F* = 3.78, *p* = 0.02), mostly after re-stimulation with *OVA* and all stress agents (*F* = 39.0 *p* < 0.0001, Fig. 6, *B* and *C*, and gating in Fig. 6, *C* and *D*). We have then tested any correlation between AID and CD40L using paired cells from 40 mice, as described for CD40L with maIl-15 and IL-1β (Fig. 2, *C* and *D*). It is interesting that two functional readings of CD40L in CD4^+^ T cells and AID in CD19^+^ B cells should be significantly correlated (*p* = 0.02, Fig. 6*E*). This raises the possibility that the stress agents may up-regulate AID in B cells through an interaction between CD40L in T cells and CD40 in B cells.

##### Correlation between the Innate and Adaptive Immunity

**FIGURE 7.**
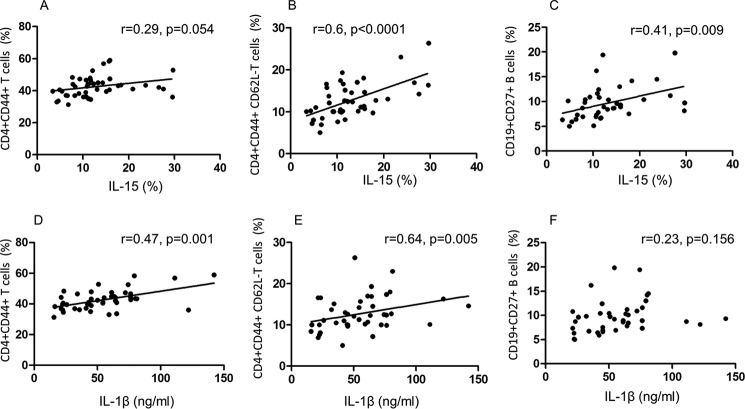
**Direct correlation between the CD4^+^ T or CD19^+^ B memory cells and IL-15 homeostatic or IL-1β inflammasome pathways.** *A–C*, significant direct correlation between IL-15 and CD4^+^CD44^+^ memory T cells (although *p* = 0.054) (*A*), CD62L^−^ effector memory T cells (*B*), and CD19^+^CD27^+^ memory B cells (*C*). *D–F*, the same cell types as *A–C*, but analysis of correlation was carried out against IL-1β; *F* failed to show significant correlation. (*n* = 40).

Any correlation between the H and I pathways of CD4^+^CD44^+^ T or CD19^+^CD27^+^ B memory cells was further explored with reference to IL-15 and IL-1β. Again, this used paired cells from 40 mice, as described above. Indeed, DC maIL-15 showed near direct correlation with CD4^+^CD44^+^ memory T cells (*p* = 0.054, Fig. 7*A*), reaching strong significance with CD44^+^CD62L^−^ effector memory T cells (*p* < 0.0001, Fig. 7*B*), as well as with CD19^+^CD27^+^ memory B cells (*p* = 0.009, Fig. 7*C*). However, significant correlation was found between IL-1β and CD4^+^CD44^+^ memory T cells (*p* = 0.007, Fig. 7*D*) and effector memory T cells (*p* = 0.005, Fig. 7*E*), but not with CD19^+^CD27^+^ memory B cells (Fig. 7*F*). The data are consistent with the present and previous finding (15) of optimum memory T cell expression requiring both H (DC-mediated maIL-15) and I (DC-mediated IL-1β) pathways, whereas memory B cells require only the H pathway.

##### The Effect of Stress Agents on OVA-specific IgG and IgA Antibodies

**FIGURE 8.**
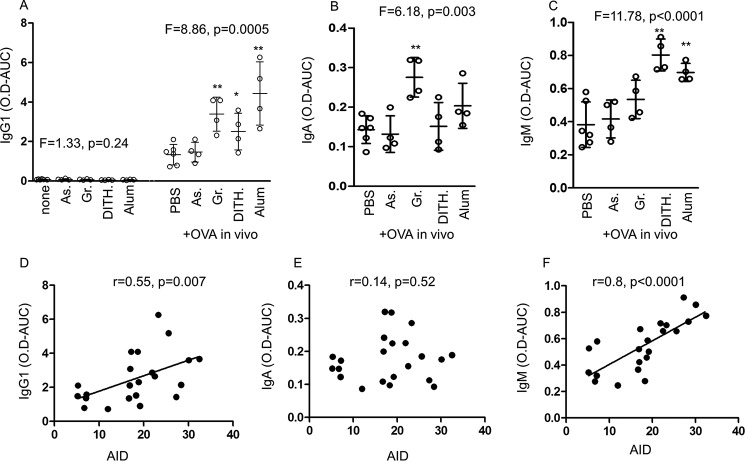
**The effect of stress agents in unimmunized and OVA-immunized BALB/c mice on IgG, IgA, and IgM antibodies to OVA and their correlation with AID.** *A–C*, antibodies to OVA assayed by ELISA showing IgG1 (*A*), IgA (*B*), and IgM (*C*) antibodies, all expressed as the total OD of the serially double diluted sera by calculating the titration area under the curve (*O.D.-AUC*). *As*, sodium arsenite; *Gr*, Gramicidin; *Dith.*, dithiocarbamate. *D–F*, direct correlation between AID and IgG1 (*D*), IgA (*E*), and IgM (*F*). All data are expressed as mean ± S.D. *, *p* < 0.05, **, *p* < 0.01; the correlations were calculated by the Spearman test.

One of the functions of AID is CSR, which elicits class switch antibody isotypes from IgM to IgG, IgA, and IgE. Here we have assayed by ELISA the IgM, IgG1, IgG2a, IgG3, and IgA antibody classes to OVA and the total concentrations of these immunoglobulins. There was little or no increase in any of the antibody classes in response to treatment with the stress agents in the unimmunized mice (Fig. 8*A*). However, in the OVA-immunized animals, IgG1-specific antibodies were significantly increased (*F* = 8.86, *p* = 0.0005) when treated with all but sodium arsenite (*p* < 0.05 to *p* < 0.001, Fig. 8*A*). IgG2a and IgG3 were not significantly affected by stress (data not shown). IgA antibodies to OVA were significantly up-regulated (*p* < 0.005; *F* = 6.18), but only with Gramicidin (*p* < 0.01; Fig. 8*B*). Surprisingly, IgM antibodies to OVA were also significantly increased (*F* = 11.78, *p* < 0.0001, Fig. 8*C*), although only to dithiocarbamate and alum (*p* < 0.005). Although the increases in IgM and IgA antibody titers were significant, they were about a tenth of those seen with IgG1 antibodies.

It is noteworthy that AID showed significant direct correlation with IgG1 (*r* = 0.551, *p* = 0.007, Fig. 8*D*) and IgM (*r* = 0.796, *p* < 0.0001, Fig. 8*F*), but not with IgA, the titer of which was only slightly increased (Fig. 8*E*). It is not clear whether SHM and/or CSR are involved, as both are mediated by AID (20), but it is likely that IgG1and especially IgA are influenced by both CSR and SHM, whereas IgM was influenced only by SHM.

## Discussion

Expansion of CD4 and CD8 T cells is essential for the survival of homeostatic immunological memory. The cytokines IL-15 and IL-7 promote proliferation of memory T cells (21, 22). This is consistent with the present findings for IL-15, although IL-7 does not appear to be up-regulated by cellular stress. IL-15 can also mediate antigen-independent proliferation of T cells by interacting with the IL-15 receptor complex (23, 24). We have demonstrated that stress-induced maIL-15/IL-15R complex interaction between DC and CD4^+^ T cells elicits homeostatic memory (9) and that this response can be independent of antigen-MHC class II T cell receptor interaction. However, stress also activates the I pathway involving caspase-1, activating IL-1β production, which interacts with IL-1βR expressed by CD4^+^ T cells. The two pathways have been induced by diverse stress agents, which elicit ROS and inducible 70-kDa heat shock protein (iHSP70). Furthermore, the H pathway activates Jak3 and STAT5 phosphorylation signaling and induces CD40L in CD4^+^ T cells, which may re-activate CD40 molecules on DC and create a feedback circuit (9). The I pathway may activate MyD88 phosphorylation. Cross-talk between the two pathways has been reported through maIL-15 and IL-1R, as soluble IL-15 with IL-7 activates the Jak1 transducer, STAT5 AKT, and PI3K signaling pathways, which may induce IL-R1 (25).

The present study deals with differences between antigen-unimmunized and OVA-immunized BALB/c mice treated with stress agents and alum. CD4^+^ T cells showed a fairly consistent pattern of responses for seven different parameters: CD40L, Tbet, IFNγ and IL-17 expression, phenotypic CD44^+^ T memory, CD44^+^ CD62L^−^ effector memory, and CD4^+^ T cell proliferation. Most of them showed up-regulation by the stress agents without OVA immunization. The specificity of these memory cells has not been determined, but they are likely to represent expansion of CD4^+^ memory T cells to multiple antigens encountered in the past. The corresponding CD4^+^ T cells immunized with OVA and treated with the stress agents showed a significantly enhanced response as compared with the unimmunized cells, and the response of the OVA-specific CD4^+^ T cells was demonstrated by the tetramer assay.

Murine transcription factors were then studied in splenic CD4^+^ T cells. Only Tbet was increased in the unimmunized mice by the stress agents, but in the OVA-immunized animals, both Tbet and RORγt were significantly up-regulated, although only if the cells were re-stimulated *in vitro* with OVA. GATA3 failed to show any significant changes. These results were corroborated with the cytokine studies showing significant enhancement of IFN-γ, without or with OVA immunization, and no increase in IL-4 or IL-10. IL-17 expression differed from RORγt in that significant increase was seen without OVA immunization, although after re-stimulation with OVA, both RORγt and IL-17 were significantly increased. The discrepancy with IL-17 might be accounted for by the earlier timing in the expression of RORγt rather than the IL-17 cytokine. Otherwise, the responses of the transcription factors to the stress agents are largely consistent with the corresponding cytokines tested and suggest that Th1 and Th17 but not Th2 responses are involved.

CD19^+^CD27^+^ memory B cells were significantly increased in the unimmunized BALB/c mice and showed 2–4-fold increase in OVA-immunized animals, when re-stimulated *in vitro* with OVA. IL-15Rα expressed by CD19^+^ or CD 27^+^ B cells was largely not affected by the stress agents, but the steady state of CD27^+^ memory B cells was 6–7-fold higher than that of the naive CD19^+^ B cells. Analysis of IL-1R1 showed similar changes, except that it increased only by 4–6-fold. These data demonstrate that although stress had no significant direct effect on the IL-15 or IL-1 receptors expressed by CD19 naive or CD27^+^ memory B cells in antigen-independent or -dependent cells (with two exceptions), both receptors were very significantly increased in memory B cells as compared with naive cells.

Turning now to functional responses, CD19^+^ B cell proliferation mostly of naive cells was significantly increased on treatment with the stress agents, as was proliferation of the OVA-dependent cells, but only if boosted *in vitro* with OVA. However, CD4^+^ T cells showed very significant increase in proliferative responses in the unimmunized and OVA-immunized animals, with further increase after *in vitro* re-stimulation with OVA. The results suggest that the memory B cells appear to be more demanding than the CD4^+^ memory T cells in OVA-dependent proliferative responses, and this might be associated with the latter utilizing both the H and I pathways, unlike memory B cells limited to the H pathway.

We have also studied AID, which mediates B cell SHM and CSR (17–19). AID in B cells failed to respond to stress without immunization but showed very significant increase when immunized and boosted with OVA. Examination of the cell surface expression of IgG subtypes as well as IgA and IgM isotypes revealed that the major IgG1 subtype was significantly up-regulated in the OVA-immunized and re-stimulated B cells with three of the four stress agents. However, IgG2a and IgG3 remained largely unchanged. The isotype switch function of CSR was evident in the increase in IgG1 and to a smaller extent with IgA antibodies. The high IgG1 antibody titers were significantly directly correlated with AID, but this was not seen with the low IgA antibody levels. This is rather surprising as IgA is dependent on CSR, and this will need to be further investigated.

A number of indices tested, such as CD40L, IFN-γ, and IL-17, as well as AID and CD-19 B cell proliferation, showed enhanced OVA-dependent responses mostly when re-stimulated *in vitro* with OVA. This was an intriguing finding, as most of these indices were significantly increased without OVA immunization. This may be accounted for under physiological conditions in which the cells are activated but in which the cells need an additional encounter with the antigen to elicit a significant response.

The critical question whether both H and I pathways are required to generate the CD44^+^ T as well as CD27^+^ B memory cells was examined by inhibition with anti-IL-15 antibodies and IL-1βRI antagonist, respectively (10, 15). Although the H pathway produces optimum CD4^+^CD44^+^ memory T cells when linked with the I pathway, the former appears to be stronger. However, with Gramicidin, neither inhibitor alone had any detectable effect, but when combined, significant inhibition resulted. Inhibition with IL-15 antibodies but not IL-1βR1 antagonist of CD19^+^CD27^+^ memory B cells suggests that only the H pathway is involved *in vivo* in mice. This difference is unlikely to be due to a lower expression of IL-1R on memory B cells as compared with T cells (about half), as some stress agents up-regulated IL-1R on B cells without affecting inhibition with IL-1β antagonist. The interaction between CD40L of CD4^+^ T cells and CD40 on CD19^+^ B cells might be involved, or alternatively the H pathway is sufficient for B cell memory stimulation. We attempted to use IL-1β receptor KO mice, but the animals we received still expressed a significant level of IL-1β, and that experiment had to be abandoned.

On the basis of the literature on homeostatic CD4^+^ T cell memory, inflammasome studies, and the present findings, we postulate that stress agents may significantly enhance immune responses *in vivo* in unimmunized and immunized mice. The mechanism may involve both the H and I pathways and result in up-regulation of CD4^+^CD45RO^+^ memory T cells, Tbet, and RORγt transcription of IFN-γ and IL-17 cytokines (15). Both the H and I pathways may be required to an unequal extent to elicit optimum responses. A feedback circuit operates in maintaining a tonic homeostatic antigen-independent and -dependent memory, predominantly by the interaction between the maIL-15 and IL-15 receptor complex inducing CD40L on CD4^+^ T cells, which bind CD40 on DC and re-activate the circuit (9). However, memory B cells *in vivo* in BALB/c mice seem to require only the H pathway. We reiterate that stress induced by a diversity of agents may drive and maintain the postulated “tonic activation” of unimmunized B memory cells (although in the past exposed to a variety of antigens) and directly immunized CD4^+^CD44^+^ T and CD19^+^CD27^+^ B memory cells. Although memory CD4^+^ T cells *in vivo* and *in vitro* require both the H and I pathways, B cells seem to be dependent *in vivo* predominantly on the H pathway.

## Experimental Procedures

### 

#### 

##### Reagents

Goat anti-mouse IL-15 antibodies were obtained from R&D (Abingdon, UK). mAb to mouse CD11c for DC, CD4, CD44, CD40L, and CD62L for T cells and mAb to mouse CD19 and CD27 for B cells were purchased from BD BioLegend (London, UK). Sodium arsenite, dithiocarbamate, and Gramicidin were obtained from Sigma-Aldrich (Dorset, UK). Alum-Gel-S, which contains 2% AL(OH)3, was obtained from Serva Electrophoresis GmbH (Heidelberg, Germany).

##### Animals and Immunization

8-week-old BALB/c mice were purchased from Charles River Laboratories and divided into 10 groups of 4–6 mice per group. Except for group 1, which was untreated, group 2 mice were injected subcutaneously with 5 μg of arsenite per mouse; group 3 mice were injected subcutaneously with 10 μg of Gramicidin; group 4 mice were injected subcutaneously with 20 μg of dithiocarbamate; and group 5 mice were injected subcutaneously with 100 μl of 10% alum-Gel-S. Groups 6–10 were immunized with 10 μg per animal OVA, and except for group 6, the above three stress agents and alum. OVA immunization was carried out subcutaneously at the base of the tail three times at 2-week intervals. 1 week after final injection, sera were collected. Mononuclear cells from the spleens were isolated and counted, and the viability of the cells was determined by the trypan blue exclusion. Animals were housed in the animal care facility at the King's College London Biological Service Department. All experiments were reviewed by the King's College London Animal Welfare and Ethical Committee and carried out under Guidance on the operation of the Animals (Scientific Procedures) Act 1986 by the UK Home office.

##### Assay of maIL-15 in Murine CD11c^+^ and CD40L in Splenic T Cells

Murine splenic CD11c^high^ DC were identified with PE-labeled anti-CD11c mAb. maIL-15 was assayed by incubating splenic cells with 5 μl (10 μg/ml) of APC-conjugated anti-mouse IL-15 mAb. To detect CD40L expression, 3 × 10^5^ splenic cells were incubated with 4 μl of PE-conjugated anti-murine CD40L and the isotype control antibody for 4 h at 37 °C. After washing, cells were labeled with CD4 or CD8 mAb. Memory cells were identified with mAb to CD44, effector cells were identified with mAb to CD62^−^, and central memory cells were identified with mAb to CD62^+^. The cells were analyzed by the BD FACSCanto II flow cytometer, using Diva software and profiles presented using WinMDI software.

##### Assays for Murine Activated Caspase-1 and IL-1β

Caspase-1 activation in mouse splenic cells was identified by using the FAM-FLICA caspase-1 kit (ABD Serotec). Murine splenic cells were incubated with 20 μl of 1:300 diluted FAM-YVAD-FMK for 1 h in 96-well round bottom plates at 37 °C. After washing, the cells were treated with PE-labeled anti-murine CD11c mAb and analyzed by flow cytometry. For IL-1β production, mouse splenic cells (100 liters of 3 × 10^6^ cells) were plated onto 96-well plates, and 10 ng/ml LPS was added. After 18 h of incubation, the supernatants were collected and IL-1 was assayed using the mouse IL-1 ELISA set (BD OptEIA^TM^)

##### CD4^+^ T and CD19^+^ B Cell Proliferative Assay

Splenic cell proliferation was determined by the Cell Trace CFSE cell proliferation kit (Molecular Probes, Invitrogen) in a 96-well plate based assay. 100 μl/well of CFSE-labeled splenic cells (2 × 10^6^/ml) were stimulated with 20 μg of OVA and various concentrations of the stress agents for 7 days. The CD4^+^ T and CD19^+^ B cell proliferation was detected with the corresponding PE-conjugated mAb. Proliferation was expressed as a proportion of proliferated cells in the total population using the CFSE dilution method.

##### Tetramer Staining Used to Detect OVA-specific CD4^+^ Cells

Splenic cells (2 × 10^6^) were incubated with 25 μl of OVA-I-Ad tetramer (5 μg/ml; National Institutes of Health, Tetramer Core Facility, Emory University, Atlanta) or the same construction of control Clip-I-Ad tetramer at 37 °C for 2 h in RPMI medium supplemented with 10% FCS in round bottom 96-well plates. Cells were then washed with the same medium and stained with FITC-anti-murine CD4 and PE-anti-murine CD44 antibodies. 20 min after incubation, cells were washed and analyzed by flow cytometry.

##### Production of Intracellular IFN-γ, IL-17, IL-4, and IL-10 in CD4^+^ T Cells

To detect cytokine production, mouse splenic cells (3 × 10^5^) were incubated with 10 μl (10 μg/ml) of APC-conjugated antibody to CD4 for 30 min. Intracellular IFN-γ, IL-4, IL-10, and IL-17 staining was carried out by incubating cells with antibodies to these cytokines following treatment of cells with fixation and permeabilization buffer (eBioscience, London, UK). In some experiments, splenic cells were stimulated *in vitro* for 18 h with 20 μg/ml OVA before detection of intracellular cytokines. After staining, the cells were washed and analyzed by the BD FACSCanto II flow cytometer, using Diva software.

##### Investigations of Transcription Factors Tbet, Gata-4, and RORγt in CD4^+^ T Cells and AID in CD19^+^ B Cells

To detect Tbet, Gata-3, and ROR(γ/t), mouse splenic cells (3 × 10^5^) were incubated with 10 μl (10 μg/ml) of APC-conjugated antibody to CD4 for 30 min. The cells were fixed and permeabilized as described as above, and transcription factors were stained with conjugated antibodies. In some experiments, splenic cells were stimulated *in vitro* for 18 h with 20 μg/ml OVA before intracellular staining. Splenic B cells were identified with antibody to CD20, and intracellular expression of AID was detected with goat anti-human/mouse AID antibodies (Santa Cruz Biotechnology). After staining, the cells were washed and analyzed as described above.

##### Detection of T and B Memory Cells

CD44 was used as a marker for CD4^+^ or CD8^+^ T cells, and CD62L was used as a marker to define effector and central memory CD4^+^ T cells. B cells were identified with CD19 mAb and memory B cell by expressing CD27. Splenic cells (3 × 10^5^) were incubated with 10 μl (10 μg/ml) of fluorochrome-conjugated antibodies for 30 min. After washing, the cells were analyzed by FACS as described above.

##### Inhibition Studies of IL-15 and IL-1β Receptor I Antagonist

To study the effect of IL-15 and/or IL-1β on the memory cell generation, splenic cells were stimulated *in vitro* for 6 days with 20 μg/ml OVA in the presence of 5 μg/ml polyclonal neutralizing antibodies to mouse IL-15 (R&D Systems) or 50 mm IL-1β receptor I antagonist (IL-1βR1, Merck) or both. Goat polyclonal antibodies were used as control. At the end of culture, memory CD44^+^ and CD62L^−^ effector memory CD4^+^ T cells and CD27^+^ memory B cells were analyzed.

##### Serum IgG Antibody Assay

Murine serum IgG antibodies to OVA were assayed by ELISA. Briefly, plates were coated with a predetermined optimal concentration of OVA (1 μg/ml) and incubated with a double dilution of serum (starting dilution of 1:100). Bound antibody was detected by incubation with rabbit IgG anti-mouse IgG (2 μg/ml; Sigma-Aldrich) antibodies, followed by affinity-purified goat anti-rabbit IgG-alkaline phosphatase conjugate (Sigma-Aldrich). OD values were determined by an ELISA reader.

##### Statistical Analysis

The statistical analyses were determined at the planning stage of the design of the investigations. The significance between groups was analyzed by ANOVA, followed by comparison with selected groups, using the GraphPad Prism 5 software. Antibody production was expressed as the total OD of the serially double diluted sera (up to 1:64,000) by calculating the titration area under the curve. For correlations, Spearman rank (non-parametric) or Pearson (parametric) correlation was used.

## Author Contributions

T. L. and Y. W. designed the experiments, analyzed data, and prepared the manuscript. Y. W. carried out the immunological assays. D. R. analyzed antibodies. M. M. was responsible for archiving plasma and cells.

## Footnotes

2 The abbreviations used are:

TCR: T cell receptor
DC: dendritic cell(s)
alum: aluminum hydroxide
ma: membrane-associated
IL-15R: IL-15 receptor
OVA: ovalbumin
H: homeostatic
I: inflammasome
ROS: reactive oxygen species
ROR: RAR-related orphan receptor
RAR: retinoic acid receptor
SHM: somatic hypermutation
CSR: class switch recombination
AID: activation-induced deamination
PE: phycoerythrin
CFSE: carboxyfluorescein succinimidyl ester
APC: allophycocyanin
ANOVA: analysis of variance
OD: optical density
FAM: 6-carboxyfluorescein.
